# Paraneoplastic pruritus presenting with Hodgkin’s lymphoma: a case report

**DOI:** 10.1186/1752-1947-8-300

**Published:** 2014-09-08

**Authors:** Jose Javier Arenas Villafranca, Marga Garrido Siles, María Casanova, Begoña Tortajada Goitia, Antonio Rueda Domínguez

**Affiliations:** 1 Pharmacy and Nutrition Service, Costa del Sol Hospital, Autovía A7, km187, CP. 29603, Marbella, Málaga, Spain; 2 Onco-hematology Service, Costa del Sol Hospital, Autovía A7, km187, CP. 29603, Marbella, Málaga, Spain

**Keywords:** Hodgkin’s Lymphoma, Paraneoplastic pruritus, Aprepitant

## Abstract

**Introduction:**

Paraneoplastic pruritus is defined as pruritus that occurs before or during the natural evolution of a hematologic disease. The reported prevalence is 30% in patients with Hodgkin’s lymphoma. The severity of this pruritus has a very negative impact on patients’ quality of life. Very few studies have been made to examine the efficacy of pharmacological treatments for this type of pruritus. One drug that appears to be effective in this respect is off-label aprepitant, a neurokinin 1 receptor antagonist.

**Case presentation:**

A 20-year-old Caucasian woman presented with lateral neck nodes, sweating, and pruritus and was diagnosed with stage IIB nodular sclerosis Hodgkin’s lymphoma. Throughout this period during the disease the pruritus was ever-present. Improvement was achieved with some of the chemotherapy treatments, but the symptom returned when the various treatments were withdrawn due to disease progression or poor tolerance. In the middle of the seventh year, she was admitted to our hospital with uncontrolled pruritus that resulted in severe lesions due to scratching. In response, aprepitant (off-label) 80mg/day was added to the chemotherapic treatment of the pruritus, after studying the various treatment options. She reported a score of 9 on a visual analogue scale for the pruritus, and a score of 3 on the Eastern Cooperative Oncology Group performance status scale of performance status. After two weeks of treatment with aprepitant, she reported a score of 5 on the visual analogue scale for the pruritus, and this improved to a score of 4 in a month, which allowed her to lead a better quality of life, with an Eastern Cooperative Oncology Group performance status score between 1 and 2.

**Conclusions:**

Several cases and case series have been reported on the use of aprepitant for paraneoplastic pruritus, but none have referred to its use for Hodgkin’s lymphoma. A prospective study was carried out to evaluate the efficacy of this drug in refractory pruritus secondary to Sezary syndrome, and other authors have studied the effectiveness of aprepitant against pruritus, secondary to biological therapy with erlotinib. In our case report, treatment was started with daily doses of aprepitant 80mg. Pruritus improvement appeared to be attributable exclusively to the administration of aprepitant.

## Introduction

Hodgkin’s lymphoma (HL) is a B-cell lymphoproliferative syndrome that is characterized by the presence of giant Reed-Sternberg cells. HL accounts for 15 to 20% of all lymphomas [1]. Although the aetiology of the process is unknown, it seems to be related to certain genetic factors and infectious agents such as the Epstein-Barr virus (EBV). The most frequent clinical manifestation (70 to 80% of cases) is the occurrence of one or more (usually painless) peripheral lymphadenopathies in supradiaphragmatic lymph node regions [2]. Between 25 and 50% of patients suffer various associated B symptoms that include weight loss, drenching night sweats, and fever. Another typical characteristic is generalized pruritus [1].

Paraneoplastic pruritus is defined as pruritus that occurs before or during the natural evolution of a hematologic disease; it is not caused by invasion of the tumor mass or by compression and disappears after removal of the tumor. The reported prevalence is 30% in patients with HL and it presents as ichthyosiform skin changes on the limbs or as eczematous lesions [3]. The severity of this pruritus has a very negative impact on patients’ quality of life [4].

Very few studies have been made to examine the efficacy of pharmacological treatments for this type of pruritus. One drug that appears to be effective in this respect is off-label aprepitant, a neurokinin 1 (NK-1) receptor antagonist that was approved in 2003 for the prevention of chemotherapy-induced nausea and vomiting, both acute and delayed. The main ligand of the NK-1 receptor, substance P, has emerged as an important mediator of the induction and maintenance of pruritus [5]. Furthermore, increased levels of NK-1 have been reported in the keratinocytes of patients with chronic pruritus [6].

In this paper, we evaluate the use of aprepitant to treat the paraneoplastic pruritus suffered by a patient with refractory HL.

## Case presentation

A 20-year-old Caucasian woman presented with lateral neck nodes, sweating, and pruritus and was diagnosed with stage IIB nodular sclerosis HL. Relevant background factors included a jaw abscess and an allergy to vancomycin. Serology revealed only positive EBV. From the first episode until her death eight years later, several treatments were applied. Firstly, chemotherapy adriamycin-bleomycin-vinblastine-dacarbazine (ABVD) for six cycles during the first seven months with complete radiological response lasting four months. Subsequent treatment involved chemotherapy gemcitabine-dexamethasone-cisplatin (GDP) for three cycles and ifosfamide-etoposide for two cycles, followed by a hematopoietic progenitor cell transplant, which achieved a complete response during the second year. Treatment continued with gemcitabine-vinorelbine for eight cycles from March to September of the third year, with a partial response, and everolimus (off-label) 5mg/day for the following 12 months, which stabilized the illness and eliminated the B "weight loss, drenching night sweats, and fever" symptoms. The following treatment was cyclophosphamide-vincristine-prednisone (CVP) for two cycles until the beginning of the fifth year of therapy, with progression. GDP for two cycles was then re-administered, followed by a cycle of bendamustine 90mg/m2, with progression in both cases. Treatment continued with gemcitabine-vinorelbine-liposomal doxorubicin (GVD, off-label) for eight cycles in the middle of the same year with a partial response and, during the sixth year, metronomic therapy with cyclophosphamide, vinblastine, and celecoxib, which stabilized the illness. She was then included in the clinical trial RV-HL-GOTEL-440 (EUDRACT: 2009-016588-12) which consists of lenalidomide and metronomic doses of cyclophophamide for refractory HL patients, but again there was disease progression, and so treatment was switched to high-dose dexamethasone (40mg/day, four days a week) in the first months of the seventh year, which obtained a partial response lasting two months. SNG-35 (brentuximab vedotin) which formed part of the laboratory’s extended-use program, was requested and five doses were administered, finishing five months later in the same year due to progression of the disease. Treatment began with oral etoposide, followed by chemotherapy with carboplatin and gemcitabine. At the beginning of the eighth year, she was admitted for palliative symptomatic treatment, beginning with thalidomide and dexamethasone, but died at the third month due to respiratory failure caused by compression of the airway by lymphoma.

Throughout this period the pruritus was ever-present. Improvement was achieved with some of the chemotherapy treatments, but the symptom returned when the drugs were withdrawn due to disease progression or poor tolerance. During the GVD treatment, the pruritus remained unchanged and during the R-HL-GOTEL-440 trial, she suffered a subjective increase in the itching sensation, which prevented sleep and caused the appearance of papulopustular lesions. Treatment with high-dose dexamethasone produced an improvement but when it was gradually withdrawn, the pruritus began to reappear.

In the middle of the seventh year, she was admitted with pancytopenia and deep vein thrombosis of the left leg, together with uncontrolled pruritus that resulted in severe lesions due to scratching. In response, aprepitant (off-label) at 80mg/day was added to the chemotherapic treatment of the pruritus, after studying the various treatment options and the scientific research available. She reported a score of 9 on a visual analogue scale (VAS) for the pruritus, and a score of 3 on the Eastern Cooperative Oncology Group (ECOG) performance status scale of performance status. After two weeks of treatment with aprepitant, she reported a score of 5 for pruritus on the VAS, and this improved to a score of 4 in a month, which allowed her to lead a better quality of life, with an ECOG level between 1 and 2. The pruritus remained controlled until she had to be readmitted, when it returned to VAS 9. Throughout this period, the lymphoma therapy remained unchanged.

Within the severe context of lymphomas, HL is generally considered to have a favorable prognosis, with 75% of patients achieving a cure if appropriate chemotherapy is received [1]. However, some cases are refractory, such as the one presented here, in which her age and quality of life led the healthcare team to consider many types of therapeutic alternatives. Thus, in this case our patient received more than 10 different treatment approaches.

Severe paraneoplastic pruritus is a symptom associated with HL for which there is no specific, effective cure, and treatment is currently based on antihistamines and corticosteroids. In the case presented, the severity of the pruritus seriously affected her quality of life and after exhausting the standard treatment alternatives, the off-label use of aprepitant was proposed in a clinical session.

Several cases and case series have been reported on the use of aprepitant for paraneoplastic pruritus, but none have referred to its use for HL. A prospective study was carried out to evaluate the efficacy of this drug in refractory pruritus secondary to Sezary syndrome, a type of non-Hodgkin’s lymphoma that primarily affects the skin and is usually secondary to mycosis fungoides. In the latter study, 80mg/day aprepitant was administered for 10 days and then on alternate days. The patients’ improvement was assessed on the Dermatology Life Quality Index (DLQI) and on a VAS. By the end of the study, a statistically significant reduction in the pruritus was reflected in both indices (*P* <0.005) and no adverse effects were detected [7]. Stander *et al*. also studied the effect of a daily dose of aprepitant 80mg/day for 3 to 13 days to treat refractory pruritus in 20 patients with various underlying pathologies. Their results also showed a significant decrease in the VAS (*P* <0.001), with a response being achieved in 80% of the patients [8].

Other authors have studied the effectiveness of aprepitant against pruritus, secondary to biological therapy with erlotinib, conducting a pilot study and administering three doses of aprepitant in a week (125mg on the first day and 80mg for the next two days). This study reported a significant decrease in the pruritus at one week after starting treatment [9]. Another paper described a case series of five patients with cutaneous T-cell lymphoma, for whom the same treatment schedule was prescribed. A response was achieved in 80% of these patients, with reductions in both the DLQI and VAS scores. Although these differences were not statistically significant, in view of the small number of patients, they were quantifiable [10].

In our case report, treatment was started with daily doses of aprepitant 80mg. This produced a marked improvement in her quality of life and in the control of the pruritus, as evidenced by the reduced score on the VAS scale, from 9 to 5 after two weeks and down to 4 after one month. This outcome improved her ECOG result, as a pruritus score of 9 on the VAS significantly affected her quality of life and forced her to remain in bed for long periods. This improvement appears to be attributable exclusively to the administration of aprepitant, as during this period the other aspects of the treatment remained unchanged.

Our patient did not present any unexpected side effects that could be related to this drug. In none of the studies reviewed was any evidence found of adverse reactions that might have resulted from the continued use of aprepitant [7-10].

## Conclusions

We report the case of a patient whose principal manifestation of HL was paraneoplastic pruritus, and how after several approaches to control it by treating HL, we finally decided to explore the off-label use of aprepitant. How aprepitant acts in this type of pruritus is not studied, but it is supposed that the main ligand of the NK-1 receptor, (substance P) is an important mediator of the induction and maintenance of pruritus, that an NK-1 receptor antagonist such as aprepitant could resolve the symptom.

According to previous publications, aprepitant has shown efficacy in controlling paraneoplastic pruritus, although to the best of our knowledge, had never been used specifically in a case of HL. More studies should be carried out to show if aprepitant could represent another option in patients with paraneoplastic pruritus whose pharmacotherapy is limited.

## Consent

Written informed consent was obtained from the patient for publication of this case report and any accompanying images. A copy of the written consent is available for review by the Editor-in-Chief of this journal.

## Abbreviations

ABVD: adriamycin-bleomycin-vinblastine-dacarbazine; CVP: cyclophosphamide-vincristine-prednisone; DLQI: Dermatology Life Quality Index; GDP: gemcitabine-dexamethasone-cisplatin; GVD: gemcitabine-vinorelbine-liposomal doxorubicin; HL: Hodgkin’s lymphoma; NK-1: neurokinin 1; VAS: visual analogue scale.

## Competing interests

The authors declare that they have no competing interests.

## Authors’ contributions

ARD and MC were the doctors in charge of the patient. MGS and BTG were the hospital pharmacists that supervised all patient treatments. JJAV was the major contributor in writing the manuscript. All authors read and approved the final manuscript.
